# Challenges in biobanking in personalized medicine – a biobank society’s view

**DOI:** 10.1186/1878-5085-5-S1-A128

**Published:** 2014-02-11

**Authors:** Hans-Peter Deigner

**Affiliations:** 1 Furtwangen University, HFU / Fraunhofer IZI, Leipzig, Germany

## 

European, Middle Eastern and African Society for Biopreservation and Biobanking (ESBB) http://www.esbb.org

Biobanks, repositories of human biospecimen (tissue, blood, bone, serum, DNA, RNA etc.) are critical resources enabling association of patients phenotypic to molecular data. Biospecimen of controlled quality thus are mandatory prerequisites for precise multiparameter diagnosis, individualized biomarker-based treatment and prognosis and therefore, are critical for the development of personalized medicine in general.

This paper will address current and future challenges and projects in biobanking including biospecimen storage conditions (standardized storage conditions or validation of storage conditions impact) in the light of changing technologies and future data analysis reflecting the fact that technological and medical knowledge evolve over time. The lack of standardization in clinical study reporting is another issue preventing annotation of pseudonymized individual molecular and clinical data and thus further analyses. Further, the importance of adequate funding, training and certification will be stressed, suggestions for biobank quality criteria presented and aspects such as quality management, biobank network extension and resource sharing discussed.

To tackle these issues, ESBB’s potential future partners and possible proposals within the new EU Program “Horizon 2020” will be introduced.

